# Validation of the DNA Methylation Landscape of TFF1/TFF2 in Gastric Cancer

**DOI:** 10.3390/cancers14225474

**Published:** 2022-11-08

**Authors:** Ze Qian, Yifan Jiang, Chunhui Shou, Jinghua Yu, Dongdong Huang, Haiyang Xie, Lin Zhou, Diyu Chen, Shusen Zheng

**Affiliations:** 1 Division of Hepatobiliary and Pancreatic Surgery, Department of Surgery, School of Medicine, First Affiliated Hospital, Zhejiang University, Hangzhou 310003, China; 2 Key Laboratory of the Diagnosis and Treatment of Organ Transplantation, Ministry of Public Health, Hangzhou 310003, China; 3 Division of Gastrointestinal Surgery, Department of Surgery, School of Medicine, First Affiliated Hospital, Zhejiang University, Hangzhou 310003, China; 4 Department of Gastroenterology, School of Medicine, First Affiliated Hospital, Zhejiang University, Hangzhou 310003, China; 5 Department of Pathology, School of Medicine, First Affiliated Hospital, Zhejiang University, Hangzhou 310003, China

**Keywords:** gastric cancer, DNA methylation, TFF1, TFF2

## Abstract

**Simple Summary:**

The members of the TFF family have been illustrated to be tumor suppressor genes in various malignancies. In this study, we first identified that TFF1/TFF2 expressions were mediated by DNA methylation in gastric cancer. Moreover, the specific CpG island sites of TFF1/TFF2, which corresponded to the downregulation of these two genes, were also discovered through integrative analysis. In addition, using the gain of function assay, it was found out that TFF1 and TFF2 could suppress the pathogenesis of gastric cancer. Totally, TFF1 and TFF2 could be the potential DNA methylation biomarkers for gastric cancer.

**Abstract:**

As one of the most frequently occurring tumor types, the increasing incidence of gastric cancer (GC) has been observed in the past decades. The recent studies have illustrated that epigenetic modifications mediated by DNA methyltransferases (DNMTs) are the major epigenetic hallmark in GC progression. Nowadays, DNA methylation was considered to be necessary for inducing the silence of tumor suppressor genes (TSGs). As an important group of peptides, the TFF family has been confirmed to function as a TSG in various kinds of cancers. However, whether TFFs could be modified by DNA methylation in gastric cancer remains unknown. Here, we initially screened out two transcriptional sequencing profiles about GC from Gene Expression Omnibus (GEO) database. The lower expression levels of TFF1 and TFF2 were observed in GC tumor tissues as compared to those in normal tissues. Additionally, utilizing the Kaplan–Meier analysis, the expressions of TFF1 and TFF2 were identified to be associated with the prognosis of GC patients. Subsequently, the integrative analysis was performed to estimate the DNA methylation level of each site in TFF1/TFF2 CpG islands. Importantly, our findings indicated that hyper-methylation of cg01886855 and cg26403416 were separately responsible for the downregulation of TFF1 and TFF2 in GC samples. In addition, utilizing the experiments in vitro, we demonstrated that TFF1/TFF2 could suppress the proliferation of GC cells. Based on these results, we suspected that TFF1/TFF2 could potentially act as the putative tumor suppressor in GC, and these two TFFs were of great value for predicting the overall survival (OS) status in the gastric cancer cohort. Totally, our findings revealed a potential therapeutic method for targeting the TFFs for the treatment of GC.

## 1. Introduction

Gastric cancer is the fifth leading malignancy with incidence and second mortality worldwide [1,2,3]. Several risk factors include salt and salt-preserved food, *H. pylori*, smoking, alcohol, and obesity [3,4,5]. At present, gastric cancer is confirmed to be a molecularly and phenotypically highly heterogeneous disease, and a series of essential cellular functions (antigrowth signaling pathways, apoptosis resistance, angiogenesis induction, and invasive or metastatic potential) are involved in the progress of this tumor [1,5,6,7]. Up to now, surgery resection is still the main treatment for gastric cancer in the early stage, which contains D2 lymphadenectomy (including lymph node stations in the perigastric mesentery and along the celiac arterial branches) [8,9]. The accumulating evidence has indicated that chemotherapy improves the survival and quality of life for patients with locally advanced unresectable or metastatic gastric cancer, but recurrence is still common [4,10]. Therefore, novel biomarkers and treatment strategies should be further explored for gastric cancer.

Presently, accumulating evidence has indicated that the progression of gastric cancer is associated with epigenetic alterations in tumor suppressor genes (TSGs) [11,12]. Though epigenetic regulations play an essential role in keeping normal biochemical functions, epigenetic aberrations also would result in harmful effects which derive from the pathogenesis of malignancies [13,14,15]. As the major method of epigenetic alterations, it has been illustrated that DNA methylation plays an essential role in various biological functions in vivo [12,16]. Several studies also demonstrated that aberrant DNA methylation was correlated with the disorders of multiple biological processes including dysregulate cell death and proliferation, developmental defects, tumor malignant progression, impaired self-renewal capacity, and immunomodulatory abnormality [17,18,19]. Thus, it is necessary to fully understand the potential contributions of DNA methylation in gastric cancer.

Trefoil factors (TFFs) are a group of stable polypeptides with a molecular weight of 6–12 kDa, which are secreted by mucus-secreting cells of the mammalian gastrointestinal epithelium. TFF1, TFF2, and TFF3 are essential components of the TFF family [20,21,22,23], which are expressed in the gastrointestinal tract and are present in virtually all mucous membrane [21,23]. Based on the special three-loop leaf-like structure, TFFs were extremely stable towards proteolytic digestion (including acid and heat degradation) [24,25]. Nowadays, TFF1 was confirmed to be necessary for the pathogenesis of breast cancer, and TFF2 was associated with the inflammatory bowel disease [22,23,26]. Although it has been shown that TFF1 and TFF2 are downregulated in primary gastric cancer [27], the interaction between TFFs and DNA methylation in gastric cancer remains unknown.

Here, we obtained two RNA-seq profiles about gastric cancer from Gene Expression Omnibus (GEO) database. Through the comprehensive analysis, the differential expressed genes (DEGs) were screened out between GC tumor tissues and their normal counterparts. Among these DEGs, we observed that two components of the TFF family (TFF1 and TFF2) were mostly downregulated in gastric cancer cases. Based on the survival analysis, the TFF1/TFF2 high-expressed cohort presented favorable overall survival (OS) and tumor-free survival (TFS) as compared to the TFF1/TFF2 low-expressed cohort. Then, we evaluated the DNA methylation level of TFF1/TFF2 CpG islands in gastric cancer, and the results indicated that the hyper-methylation of cg01886855 (TFF1-MS) was responsible for the suppression of TFF1 in the tumor, and cg26403416 (TFF2-MS) was correlated with the methylation of TFF2 in gastric cancer. In addition, we found out that the proliferative ability was suppressed followed by the mutation of TFF1-MS and TFF2-MS in tumor cells. Totally, we considered that TFF1 and TFF2 could be the potential DNA methylation biomarkers for gastric cancer.

## 2. Methods and Materials

### 2.1. Cell Culture

The gastric cancer cell lines MNK-1 and AGS were purchased from the Cell Bank of Type Culture Collection of the Chinese Academy of Sciences, The Shanghai Institute of Cell Biology, and The Chinese Academy of Sciences. AGS were cultured in F12 (Cat. No. CM-0022, Procell, Hangzhou, China) containing 10% fetal bovine serum (Cat. No. A3160802, Gibco, Mexico City, Mexico) and MNK-1 was cultured in 1640 (Cat. No. SH30026.01B, HyClone, Los Angeles, America) containing 10% fetal bovine serum (Cat. No. A3160802, Gibco, Mexico City, Mexico). The cell cultivation was conducted in a 37 °C, 5% CO_2_ humidified incubator.

### 2.2. Data Sources

In this study, two gene expression datasets about gastric cancer were obtained from the GEO database (https://www.ncbi.nlm.nih.gov/geo/) (accessed on 1 February 2022). A total of 1589 series, which were associated with human gastric cancer were retrieved from the database. After a careful review, specific gene expression profiles namely, GSE37023 and GSE26899 were selected. All of the data utilized in the study is freely available online, and no animal or human experimentation was associated with this study.

### 2.3. Data Processing of DEGs

GEO2R online analysis tool in NCBI (https://www.ncbi.nlm.nih.gov/geo/geo2r/)(accessed on 1 February 2022), was used to analyze the differential genes between tumor and normal tissues. The adjusted *p*-value and |logFC| were calculated. The differential gene was considered to meet cutoff standard requirements with adjusted *p* < 0.05 and |logFC| ≥ 2.0. Statistical analysis was carried out for each dataset. The web tool (bioinformatics.psb.ugent.be/webtools/Venn/) was used to obtain the Venn diagram.

### 2.4. GO and KEGG Pathway Analysis of DEGs

We carried out the Gene Ontology (GO) annotation analysis and KEGG pathway enrichment analysis of DEGs via the Database for Annotation, Visualization and Integrated Discovery (DAVID) tools (https://david.ncifcrf.gov/) (accessed on 1 February 2022). *p* < 0.01 and gene counts ≥10 were considered statistically significant.

### 2.5. PPI Network Construction

Search Tool for the Retrieval of Interacting Genes (STRING) was used to obtain a PPI map of DEGs. We extracted the PPI pairs whose combine score >0.4, and Cytoscape software (www.cytoscape.org/) (accessed on 1 February 2022), were used to visualize the PPI network. We considered the top 10 genes in the central index as the core candidate genes.

### 2.6. RNA Isolation and Quantitative Real-Time PCR (qRT- PCR)

TRIzol reagent (TaKaRa, Beijing, China) was used to isolate total RNAs. PrimeScript RT Reagent Kit (TaKaRa, Beijing, China) was used to construct cDNA library. SYBR Green PCR Kit (Takara, Beijing, China) and ABI 7500 FAST Real-Time PCR system (Applied Biosystems, Waltham, MA, USA) were used for the quantitative real-time PCR analysis. The 2ΔΔCt method was used for relative quantification of mRNA expression, and the quantification was completed after the data were normalized with respect to GAPDH levels which was considered as the endogenous reference.

### 2.7. Construction of the Mutation Vectors for TFF1/TTF2 DNA Methylation Sites

Firstly, according to the DNA methylation sequencing data from TCGA, we identified that TFF1 and TFF2 were hyper-methylated in gastric cancer. Then, we comprehensively analyzed the CpG islands of TFF1 and TFF2 in gastric cancer tissues, and the results indicated that cg01886835 and cg26403416 were responsible for the methylation of TFF1 and TFF2, respectively, in gastric cancer. Combined with the 3′-UTR sequencing of these two sites from UCSC web tool, the core section of these two sites was obtained (1500–2000 bp). Subsequently, we further transformed CG into AG and constructed TFF1/TFF2 mutant plasmids. All the wild-type and mutation sites of cg01886835 and cg26403416 were shown in Figure S4.

### 2.8. Double Luciferase Report Assay

For the Luciferase assay, we initially dispensed 100 µL of Luciferase Assay Reagent II for each sample into a white Optiplate 96 (PerkinElmer, Waltham, MA, USA), and then 20 µL of lysed product was supplied into each sample. Meanwhile, the plate was read in a luminometer (Tecan Infinite 200), which is programmed to perform a 12 s measurement read for Firefly Luciferase activity. Subsequently, we added 100 µL of 1× Stop & Glo Reagent in each mixture, and the plate was read again with a 12 s measurement for Renilla Luciferase activity.

### 2.9. Cell Viability Assay and Ethynyl Deoxyuridine (EdU) Assay

CCK-8 assay: The proliferation assays were carried out by seeding gastric cancer cells in 96-well plates (1200 cells/well). Cell growth and viability were determined by measuring the absorbance of the samples at 450 nm with the help of the Cell Counting Kit-8 (CCK-8) (Dojindo). After specific days of cultivation, 10 µL CCK-8 reagent was added to each well followed by culturing for 2 h at 37 °C in 5% CO_2_. The absorbance at 450 nm was measured using a microplate reader. EdU assay: Gastric cancer cells (2 × 10^5^) were plated in 24-well plates and incubated for 24 h. The EdU assays were performed with a 5-ethynyl-2′-deoxyuridine (EdU) cell proliferation assay kit (Cat. no. C6016S; UElandy, Hangzhou, China). 0.1 mL of 50 µM EdU was added into each well of 500 mL medium for 2 h. Then, we fixed cells with 4% polyformaldehyde in PBS at room temperature for 30 min and subsequently incubated with Apollo staining solution and Hoechst 33342 for 30 min. Fluorescence microscopy was performed.

### 2.10. Statistical Analysis

The SPSS 22.0 software (IBM Corp., Armonk, NY, USA) was used for statistical analyses. Student–Newman–Keuls test was used as a post hoc test to compare the variances from multiple groups. Student’s *t*-test comparisons was used to compare the variances from two groups. The correlation of genes with the overall survival was analyzed with the Kaplan–Meier analysis. Data were presented as the mean ± standard deviation. *p* < 0.05 was considered to indicate a statistically significant difference.

## 3. Result

### 3.1. Identification of the Differential Expression Genes in Gastric Cancer

**Figure 1:**
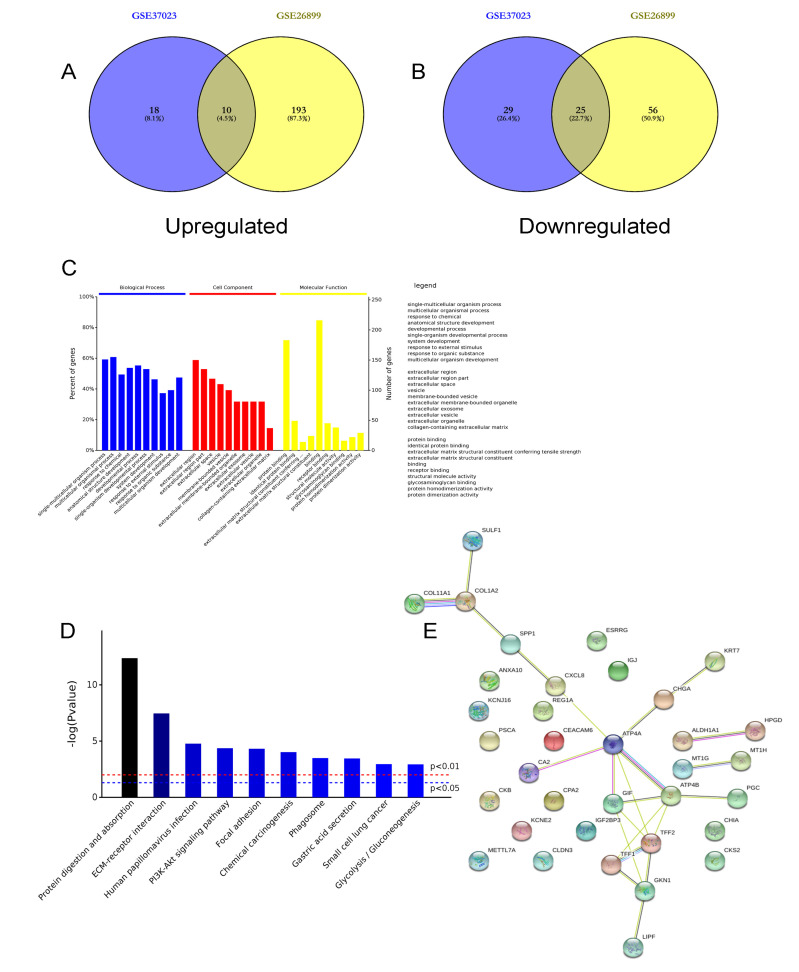
Identifying the differential expression genes in gastric cancer. Using two GEO datasets (GSE37023 and GSE26899), we observed that, both in these two expression profiles, 10 genes were upregulated (**A**) and 25 genes were downregulated (**B**) in gastric cancer as compared to normal tissues. Then, the GO analysis (**C**) and KEGG enrichment analysis (**D**) of these 35 DEGs were then carried out. (**E**) Through the web tool called Cytoscape, we established the PPI network, and ATP4A, ATP4B, TFF2, GIF, GKN1, COL1A2, TFF1, CHGA, SPP1, and CXCL8 were considered the 10 top genes among the DEGs.

Initially, two expression profiles about gastric cancer were collected from the GEO database (GSE37023 and GSE26899). Through the integrative analysis, we identified the DEGs between the tumor samples and their noncancerous counterparts. Consistently, the significant criteria were set (*p* < 0.05 and | log fold change [FC]| ≥2), and both in these two expression profiles, we explored 10 upregulated genes and 25 downregulated genes in gastric cancer as compared to normal specimens (Figure 1A,B). Utilized by the DAVID web tool, we carried out the KEGG enrichment and GO function assessment among these DEGs. From the results of GO function analysis, we found out that these DEGs were involved in the biological process (BP) (including single-multicellular organism process, multicellular organismal process, and response to chemical), cell component (CC) (including extracellular region, extracellular space, and membrane-bounded vesicle), and molecular function (MF) (including protein binding and receptor binding) (Figure 1C). As shown in Figure 1D, the results of the KEGG enrichment analysis indicated that the DEGs were mainly enriched in protein digestion and absorption, ECM–receptor interaction, human papillomavirus infection, and PI3K-AKT signaling pathway. Subsequently, the consistent protein–protein interaction (PPI) analysis was conducted to estimate the core genes among these DEGs. Utilized by Cytoscape, the PPI results were visualized (Figure 1E). Then ATP4A, ATP4B, TFF2, GIF, GKN1, COL1A2, TFF1, CHGA, SPP1, and CXCL8 were considered as the 10 top genes in the DEGs, which were the potential targets for the subsequent experiments.

### 3.2. Downregulation of TFFs Indicated the Poor Prognosis in Gastric Cancer Patients

**Figure 2:**
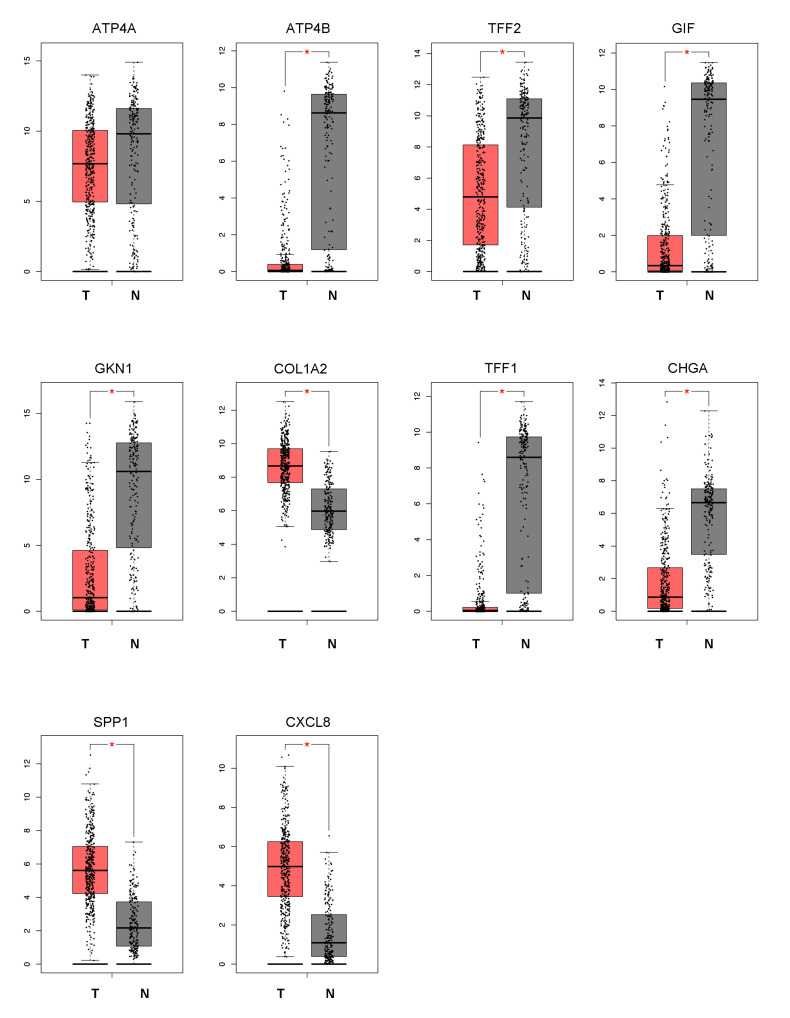
The expression patterns of 10 hub genes in gastric cancer. Utilizing the GEPIA database, we evaluated the expression patterns of these 10 hub genes in gastric cancer, and the results demonstrated that ATP4B, TFF2, GIF, GKN1, and TFF1 were significantly downregulated in tumor samples compared with the normal samples. (* *p* < 0.05, T = tumor tissue, N = normal tissue).

**Figure 3:**
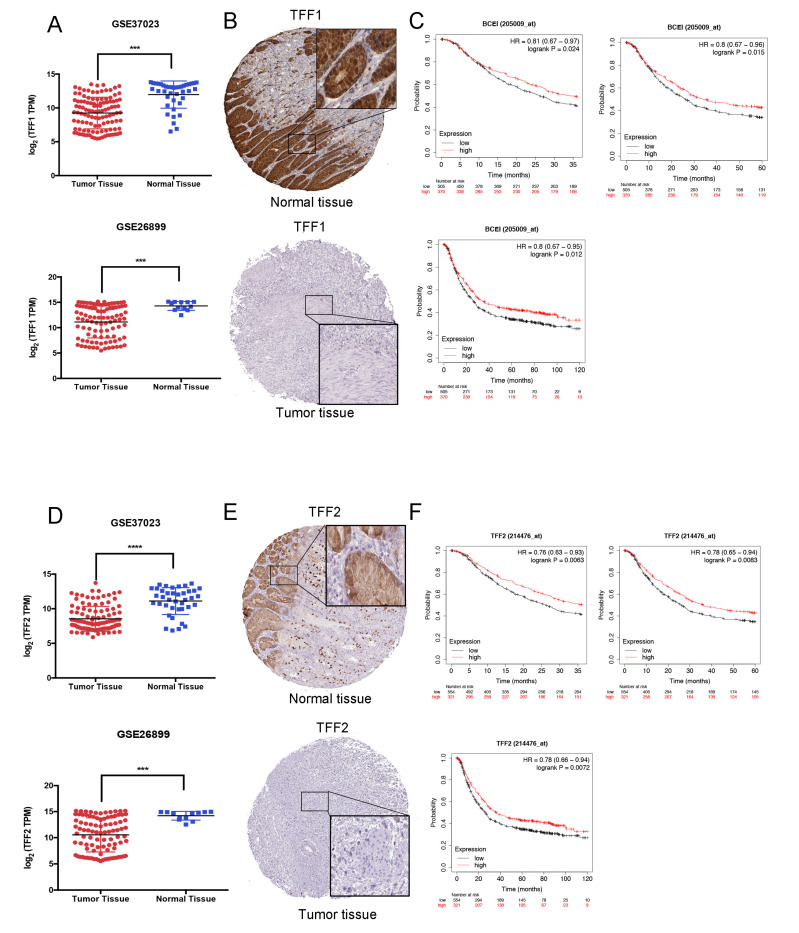
TFFs could potentially act as the suppressor genes in gastric cancer. In the GSE37023 and GSE26899 datasets, the mRNA expression of TFF1/TFF2 was upregulated in normal gastric tissues as compared to tumor tissues(**A**,**D**), and the IHC results also indicated that the normal tissues showed higher protein level of TFF1/TFF2 than gastric cancers (**B**,**E**). In addition, based on the Cancer Genome Atlas (TCGA) database, the lower expression of TFF1/TFF2 predicted the poorer 3-year, 5-year, and 10-year OS in gastric cancer patients (**C**,**F**). (*** *p* < 0.001, **** *p* < 0.0001).

Recent studies indicated that the silence of tumor suppressor genes (TSGs) could be mediated by DNA methylation in different malignancies, and these TSGs could potentially be the candidates of DNA methylation biomarkers for the tumor patients [28,29,30]. Based on the GEPIA web tool, we examined the expressions of these 10 genes in gastric cancer specimens from TCGA. Compared with the normal samples, the results demonstrated that ATP4B, TFF2, GIF, GKN1, and TFF2 were low expressed in tumor samples, suggesting these targets might act as the TSGs in gastric cancer (Figure 2). Interestingly, it has to be mentioned that TFF1 and TFF2 belonged to the TFF family, which are specifically expressed in the gastrointestinal tract and are present in virtually all mucous membranes. To determine the clinical relevance of the key down-regulated genes in the gastric cancer cohort, we performed a Kaplan–Meier survival analysis and observed that patients with lower TFF1 and TFF2 levels owned shorter overall survival (OS) and tumor-free survival (TFS) time (Figures S1 and S2). In addition, we further examined the expression of TFFs in two GEO expression profiles. As Figure 3A showed, the mRNA level of TFF1 was reduced in tumor specimens as compared to adjacent normal tissues. The subsequent immune histochemical (IHC) staining assay was also conducted, and the protein level of TFF1 between the tumor and normal tissues was detected. Our results revealed that the protein expression of TFF1 was also suppressed in gastric cancer specimens (Figure 3B).

Then, we evaluated the relationship between the TFF1 expression and prognosis in gastric cancer cohort. Visualized by UCSC-XENA, we found out that the lower expression of TFF1 predicted the better overall survival status of the gastric cancer patients (Figure 3C). Similarly, we discovered that the mRNA and protein expression of TFF2 were downregulated in tumor tissues and TFF2 was associated with the prognosis of gastric cancer patients based on TCGA database (Figure 3D–F). Totally, all these results highlighted the clinical significance of TFFs in gastric cancer and support the idea that TFF1 and TFF2 could be the potential biomarkers for the gastric cancer diagnosis and prognosis.

### 3.3. TFFs Could Be Modified by DNA Methylation in Gastric Cancer

**Figure 4:**
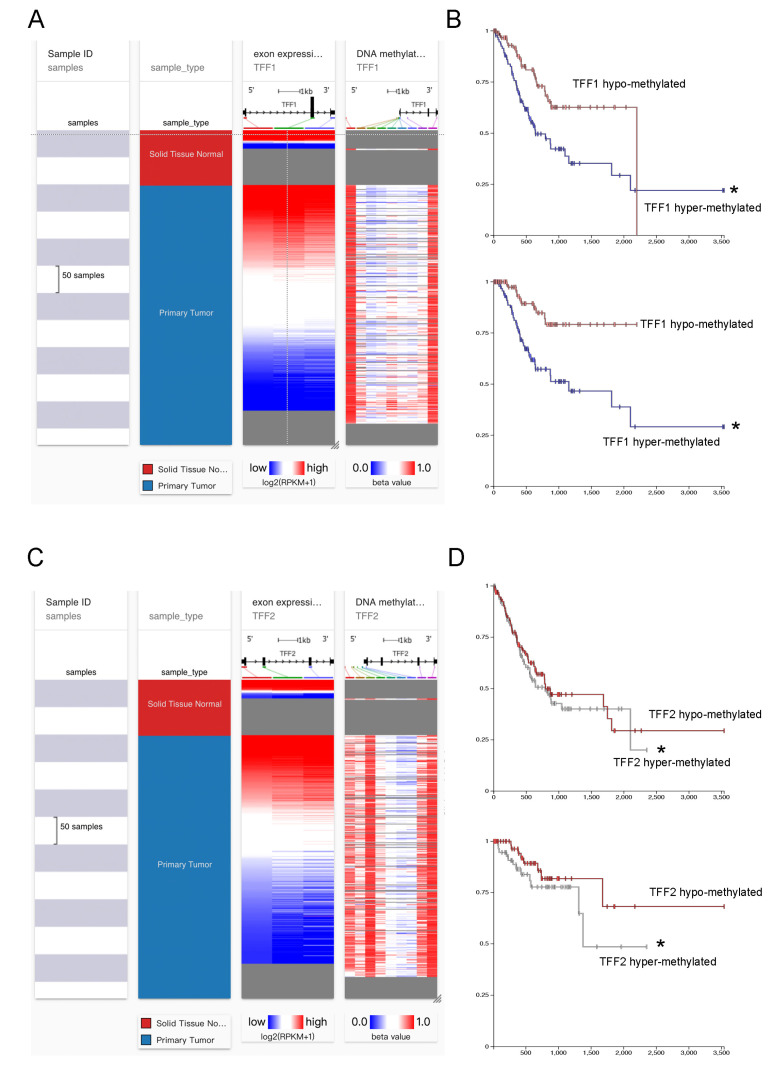
TFF1/TFF2 could be modified by DNA methylation in gastric cancer. (**A**,**B**) Through the UCSC-XENA database, it was observed that the gastric cancer tissues presented lower TFF1 and TFF2 expressions than normal tissues, and their mRNA expression was negatively correlated with the DNA methylation level. (**C**) Higher TFF1 DNA methylation levels predicted a worse prognosis for gastric cancer patients. (**D**) Similarly, the low TFF2 DNA methylation cohort showed a better prognosis than high TFF2 DNA methylation cohort. (* *p* < 0.05).

Cancer initiation is suggested to be influenced by both epigenetic and genetic events, and recent evidence has ensured that DNA methylation functioned as essential targets for tumor development. Here, we suspected whether TFF1/TFF2 could be modified by DNA methylation. It was observed that the gastric cancer tissues presented lower TFF1 and TFF2 expressions than normal tissues, and their mRNA expression was negatively related to the DNA methylation level (Figure 4A,B). Then, we also validated the impact of TFFs DNA methylation levels on the prognosis of GC patients. As Figure 4C shows, a higher TFF1 DNA methylation level predicted a worse prognosis for the patients. Similarly, the low TFF2 DNA methylation cohort showed a better prognosis than the high TFF2 DNA methylation cohort (Figure 4D). All these results demonstrated that TFFs could potentially be DNA methylated in gastric cancer.

### 3.4. Identification of the Specific CpG Island Site for TFF1 and TFF2

**Figure 5:**
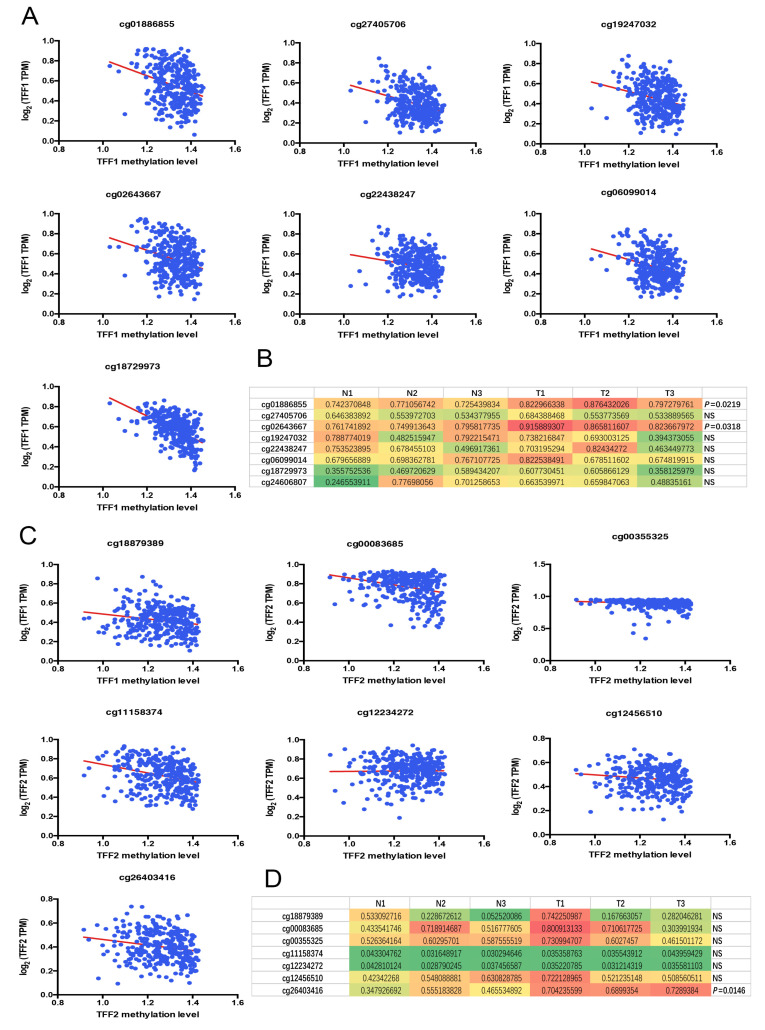
Identification of the specific CpG island site for TFF1 and TFF2 in gastric cancer. Firstly, we analyzed methylated DNA immunoprecipitation (MeDIP) sequencing available from our center and Cancer Genome Atlas (TCGA). The results indicated that, both in the MeDIP sequencing data from TCGA (**A**) and our center (**B**), tumor tissues exhibited higher DNA methylation levels in TFF1 CpG island in cg01886855 and cg02643667. Meanwhile, the TFF2 CpG island site cg26403416 (TFF2 MS) presented higher DNA methylation levels in cancer than normal specimens (**C**,**D**). (NS = No significance).

Through the comprehensive analysis mentioned above, we initially confirmed that high DNA methylation could lead to the downregulation of TFF1/TFF2 in gastric cancer tissues. Next, in order to identify methylation sites in the TFF1 and TFF2 CpG island, the Meth Primer was used in the subsequent study. According to the analysis results, it was found out that the several CpG island sites of these two genes were detected. Therefore, we performed the in vitro experiments to discover the specific CpG island sites which triggered TFF1 and TFF2 DNA methylation. Firstly, we analyzed methylated DNA immunoprecipitation (MeDIP) sequencing obtained from TCGA and our hospital. The results indicated that the TFF1 CpG island in cg01886855 and cg02643667 were highly methylated in tumor samples (Figure 5A,B). Meanwhile, the TFF2 CpG island site cg26403416 (TFF2 MS) presented higher DNA methylation levels in cancer than normal specimens (Figure 5C,D).

**Figure 6:**
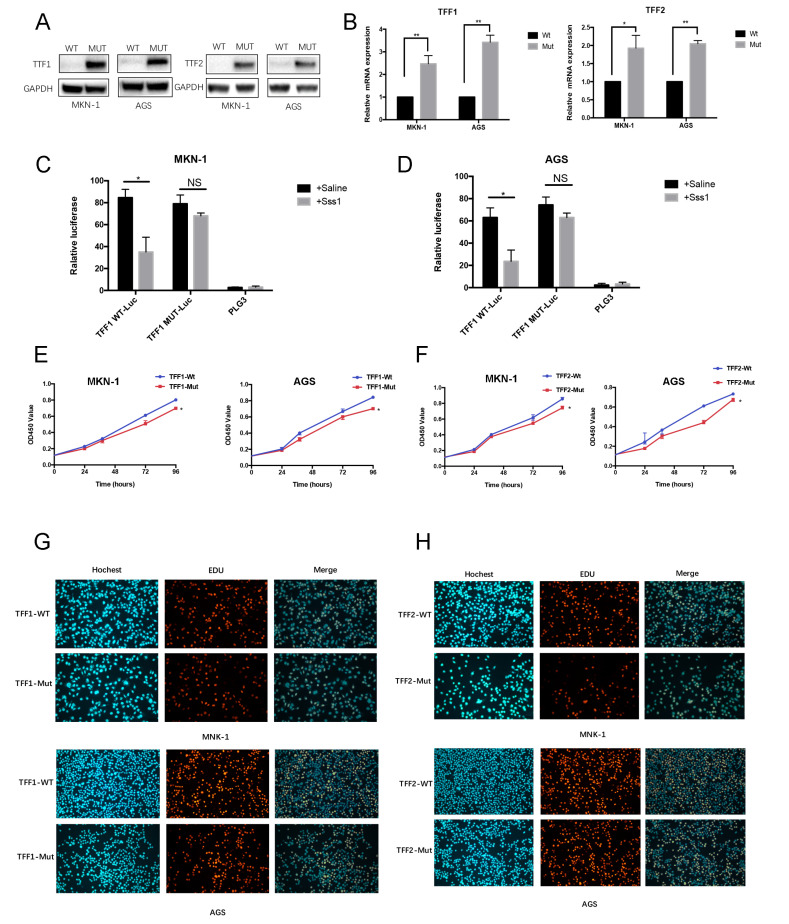
Mutation of TFF1/TFF MS led to the reduced proliferative abilities of gastric cancer cells. After transfecting the wild-type (WT) and mutation-type (MUT) plasmids into gastric cancer cell lines, the protein and mRNA expression were reverted in two gastric cancer cell lines followed by the mutations of TFFs (**A**,**B**). Then we transfected these constructs into tumor cells and revealed a marked increment of luciferase activity in the TFF1/TFF2 MUT group compared with the TFF1/TFF2 WT group (**C**,**D**). Performing the CCK-8 assay and EdU staining assay, the results revealed that the mutations of TFF1/TFF2 MS led to reduced proliferation ability in MNK-1 and AGS cells (**E**–**H**). (* *p* < 0.05, ** *p* < 0.01).

Furthermore, wild-type (WT) and mutation-type (MUT) plasmids of TFF1 MS and TFF2 MS were constructed to evaluate the specific function of these two methylation sites in GC. Then, we transfected these constructors into gastric cancer cell lines. Following the TFF1/TFF2 MS mutations, the protein and mRNA expression were reverted in two GC cell lines (MNK-1 and AGS, shown in Figure 6A,B). Then, we observed a marked increment of luciferase activity in the TFF1/TFF2 MUT group compared with the TFF1/TFF2 WT group (Figure 6C,D). Additionally, CCK-8 assay and EdU staining assay were performed to examine the contributions of TFF1/TFF2 MS in GC cells. As shown in Figure 6E–G, the MUT of TFF1/TFF2 MS led to reduced proliferation ability in tumorous cells. These results demonstrated that TFF1/TFF2 MS were the specific CpG island site separately for the TFF1/TFF2 DNA methylation in gastric cancer.

### 3.5. DNMT1 Regulated the TFFs DNA Methylation in Gastric Cancer

**Figure 7:**
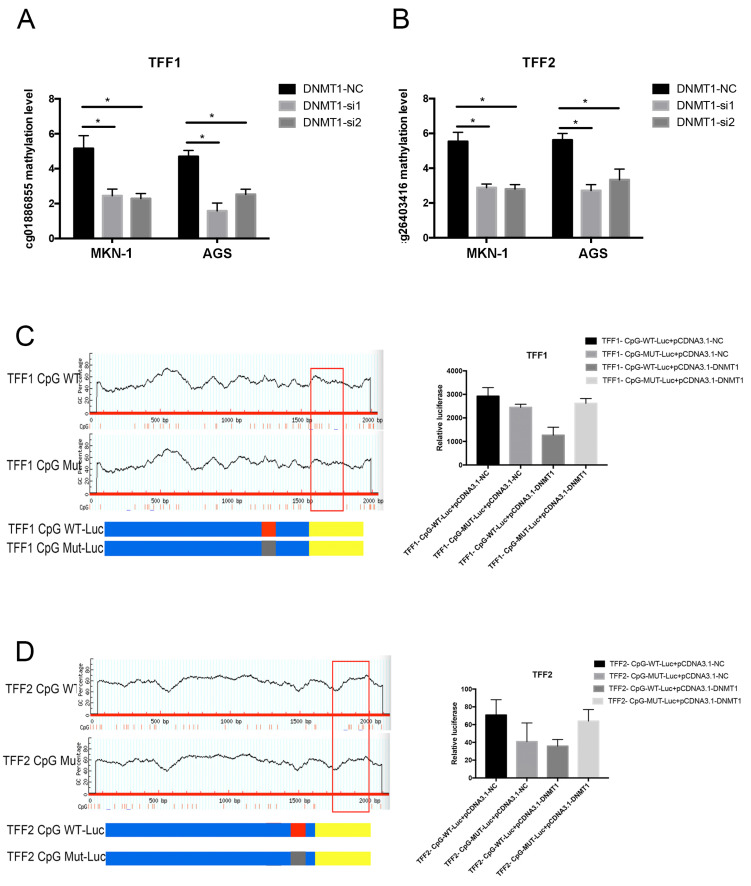
DNMT1 was necessary for the DNA methylation of TFFs in gastric cancer. The TFF1 DNA methylation level of cg01886855 (TFF1 MS) site was reduced followed by the knockdown of DNMT1 (**A**). In addition, the downregulation of DNMT1 induced the low DNA methylation level of cg2406316 of TFF2 in tumor cells (**B**). The interactions between TFF1/TFF2 and DNMT1 were examined and studied by the use of luciferase double report assay (**C**,**D**). (* *p* < 0.05).

Up to now, many studies have indicated that DNA methyltransferase (DNMTs) might be the necessary components for the DNA methylation process. Therefore, we tried to detect whether the methylation level of TFFs is mediated by the DNMTs. Through the Starbase database, the mRNA expression of TFF1/TFF2 was negatively correlated to the expression of DNMT1, DNMT3A, and DNMT3B (three key types of DNA methyltransferase, Figure S3A,B). Interestingly, we observed that only the silence of DNMT1 led to the upregulation of TFF1/TFF2 in GC cells, but not the DNMT3A and DNMT3B (Figure S3C,D). Additionally, the expression level of cg01886855 (TFF1 MS), which is the regulatory site of DNA methylation responded to TFF1, was reduced after knockdown of DNMT1 (Figure 7A,B). In addition, the downregulation DNMT1 induced the low DNA methylation level of cg2406316 of TFF2 in tumor cells. The interactions between TFF1/TFF2 and DNMT1 were examined and studied by the use of luciferase double report assay (Figure 7C,D). The results revealed that the DNA methylation level of TFF1/TFF2 MS were regulated by the DNMT1.

## 4. Discussion

Gastric cancer (GC) is frequently occurred in Eastern Asian countries and has become the third most common cause of cancer death globally. According to its highly heterogeneous characteristics, the pathogenesis of gastric cancer remains poorly understood [10,31]. At present, many risk factors such as alt-preserved food, smoking, and alcohol have been identified [7,8,9]. Recent studies have classified gastric cancer into early and non-early stage [5,6]. As for patients in the early stage, endoscopic resection is the main treatment [3,4]. For the non-early, operable gastric cancer is treated with surgery. However, the therapeutic effect of chemotherapy is limited for the patients in advanced stage [1,2]. Thus, in this study, we tried to explore novel biomarkers for gastric cancer.

Initially, we collected two RNA-seq data about gastric cancer from the GEO database. Comparing with the adjacent normal tissues, it was identified that TFF1 and TFF2 were both downregulated in tumor tissues, which is similar to the previous studies [27] Through the Kaplan–Meier survival analysis based on TCGA database, we found out that high TFF1/TFF2 expressed cohort showed an advantage in OS and TFS as compared to TFF1/TFF2 low expressed cohort. Thus, we suspected that TFFs could function as tumor suppressor genes (TSGs) to inhibit the development and progression of gastric cancer. Reviewing the previous research, TFFs expressions are reported to be mediated by epigenetic modifications, which may be correlated with H. pylori-infected gastric carcinoma [32,33]. Additionally, the loss of TFF1 expression leads to a cascade of gastric lesions, including low-degree dysplasia, high-degree dysplasia, and adenocarcinoma [34]. This suggests that TFF1 and TFF2 play important roles in gastric cancer tumorigenesis and progression.

In mammals, as the most intensely studied epigenetic modification, DNA methylation promotes gene expression and stable gene silence. Many studies have confirmed that DNA methylation is directly associated with the downregulation of TSGs in malignancies. It is commonly known that the inactivation of certain TSGs occurs as a consequence of hyper-methylation within the promoter regions. Furthermore, hypermethylated genes have recently gained increasing attention as biomarkers for the diagnosis and treatment of patients. Therefore, it is encouraging for us to explore the importance of DNA methylation alterations in gastric cancer.

Utilizing the UCSC XENA database, we tried to discover the potential correlation between DNA methylation and TFF1/TFF2 expressions. Through the integrative analysis, it was observed that TFF1 and TFF2 were low expressed in tumor specimens, and their mRNA expressions were negatively associated with the DNA methylation level. In addition, we divided the GC cases from TCGA data based into low TFF1/TFF2 DNA methylation group and high TFF1/TFF2 DNA methylation group. It was indicated that, following the hyper-methylation of TFF1/TFF2, the 1-OS, 3OS-, and 5-OS became worse in GC patients. Next, Meth Primer was used to study and identify methylation sites in the TFF1 and TFF2 CpG islands. Through analyzing the methylated DNA immunoprecipitation (MeDIP) sequencing profiles available from our center and The Cancer Genome Atlas (TCGA), we identified that cg01886855 (TFF1 MS) and cg26403416 (TFF2 MS) might be the key CpG island sites separately for the TFF1/TFF2 DNA methylation.

In order to discover the function of TFF1 MS and TFF2 MS in gastric cancer, wild-type (WT) and mutation-type (MUT) plasmids were constructed. Then, we transfected these constructors into gastric cancer cell lines. Followed by the mutation of TFF1 MS and TFF2 MS, the protein and mRNA expression were reverted in two gastric cancer cell lines. Carrying out the CCK-8 assay and EdU staining assay, it was found that the mutations of TFF1/TFF2 MS led to reduced proliferation ability in tumorous cells. These results demonstrated that TFF1/TFF2 MS were the specific CpG island site separately for the TFF1/TFF2 DNA methylation in gastric cancer. In addition, through the subsequent analysis, we illustrated that the DNA methylation level of these two sites was regulated by the DNMT1.

## 5. Conclusions

Taken together, our findings suggested that TFF1 and TFF2 were both downregulated in gastric cancer, and their mRNA expressions were silenced by DNA methylation. Through the experiments in vitro, hyper-methylation of TFF1/TFF2 would facilitate the proliferation of GC cells, indicating that TFFs played a crucial role in GC pathogenesis. Furthermore, we also identified TFFs were also related to the prognosis in GC patients. Therefore, we considered that TFF1/TFF2 might be the potential DNA methylation target for gastric cancer. More studies are necessary for further investigation of TFF1/TFF2 functions.

## Data Availability

The original contributions presented in the study are included in the article/Supplementary Material.

## Supplementary Materials

The following supporting information can be downloaded at: https://www.mdpi.com/article/10.3390/cancers14225474/s1.
