# Supplementary material for: Validation of the DNA Methylation Landscape of TFF1/TFF2 in Gastric Cancer
Source: Cancers (Basel). 2022 Nov 8;14(22):5474. doi: 10.3390/cancers14225474 (PMC9688599; doi:10.3390/cancers14225474)
Supplement: Supplementary file 1 [file cancers-14-05474-s001.zip › cancers-1966095-supplementary.pdf]

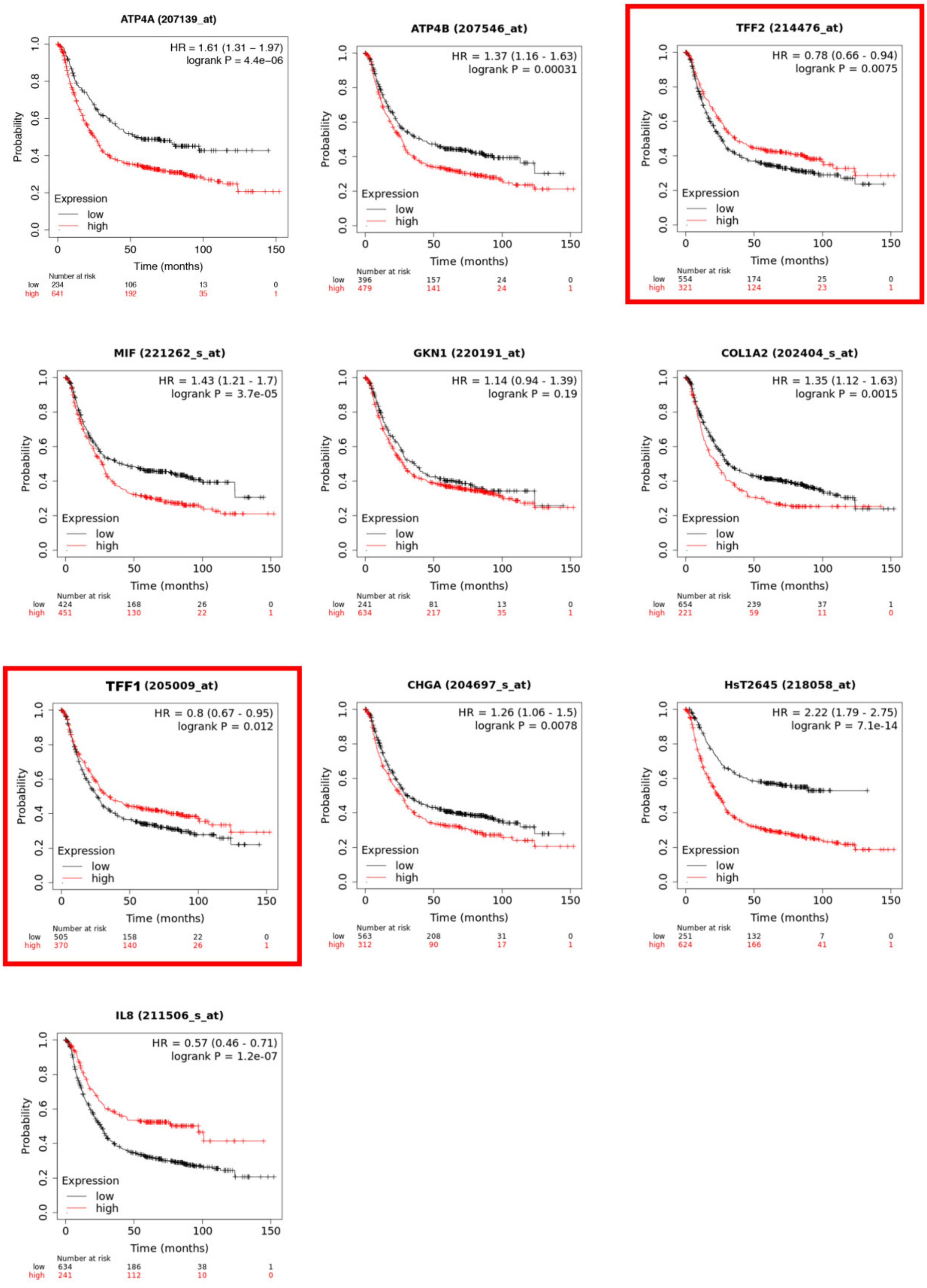

Figure S1 The Kaplan-Meier survival analysis was performed and it was observed that lower levels of TFF1 and TFF2 indicated the shorter overall (OS) time in the gastric cancer patients.

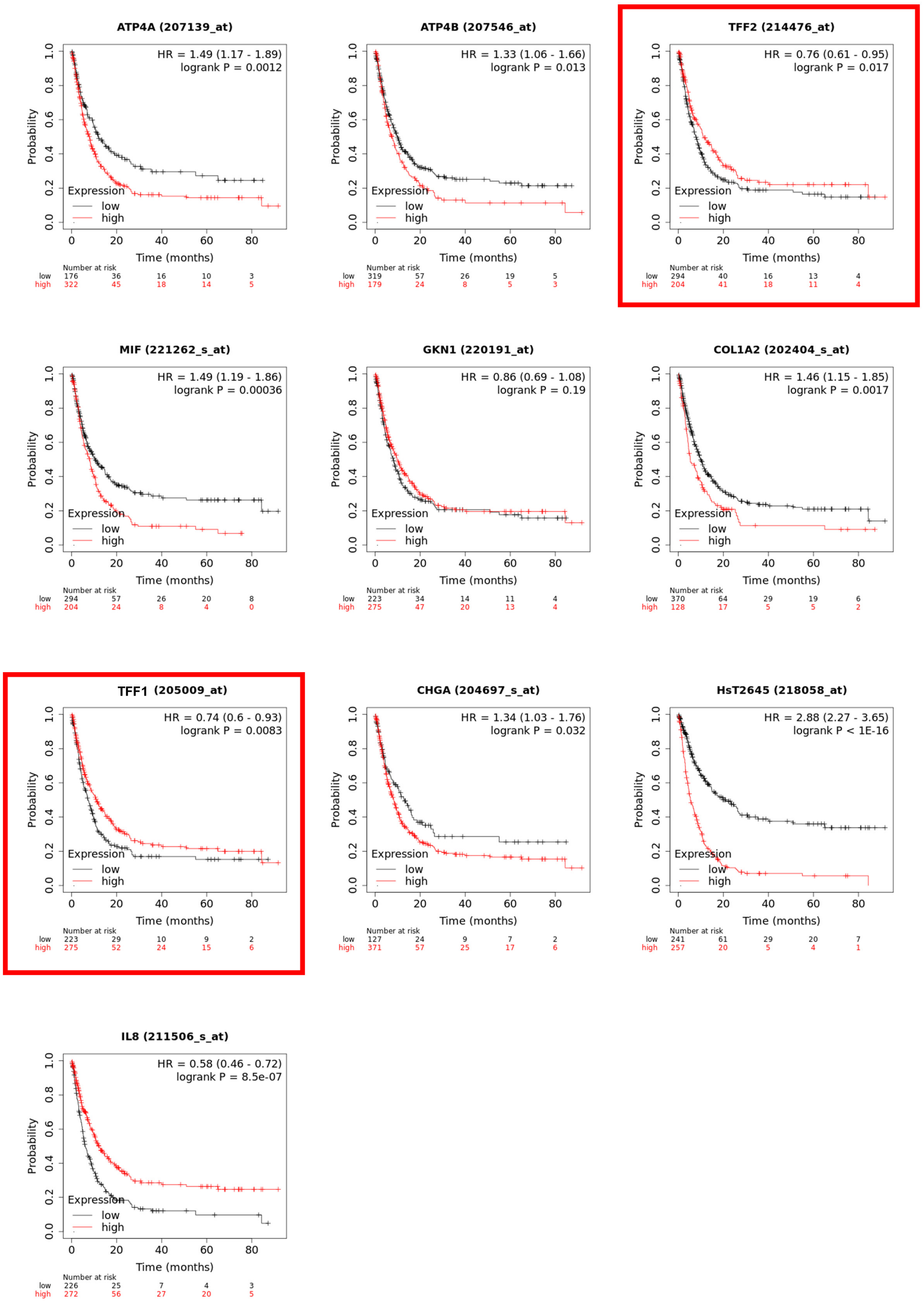

Figure S2 The Kaplan-Meier survival analysis was performed and it was observed that lower levels of TFF1 and TFF2 indicated the shorter tumor free survival (TFS) time in the gastric cancer patients.

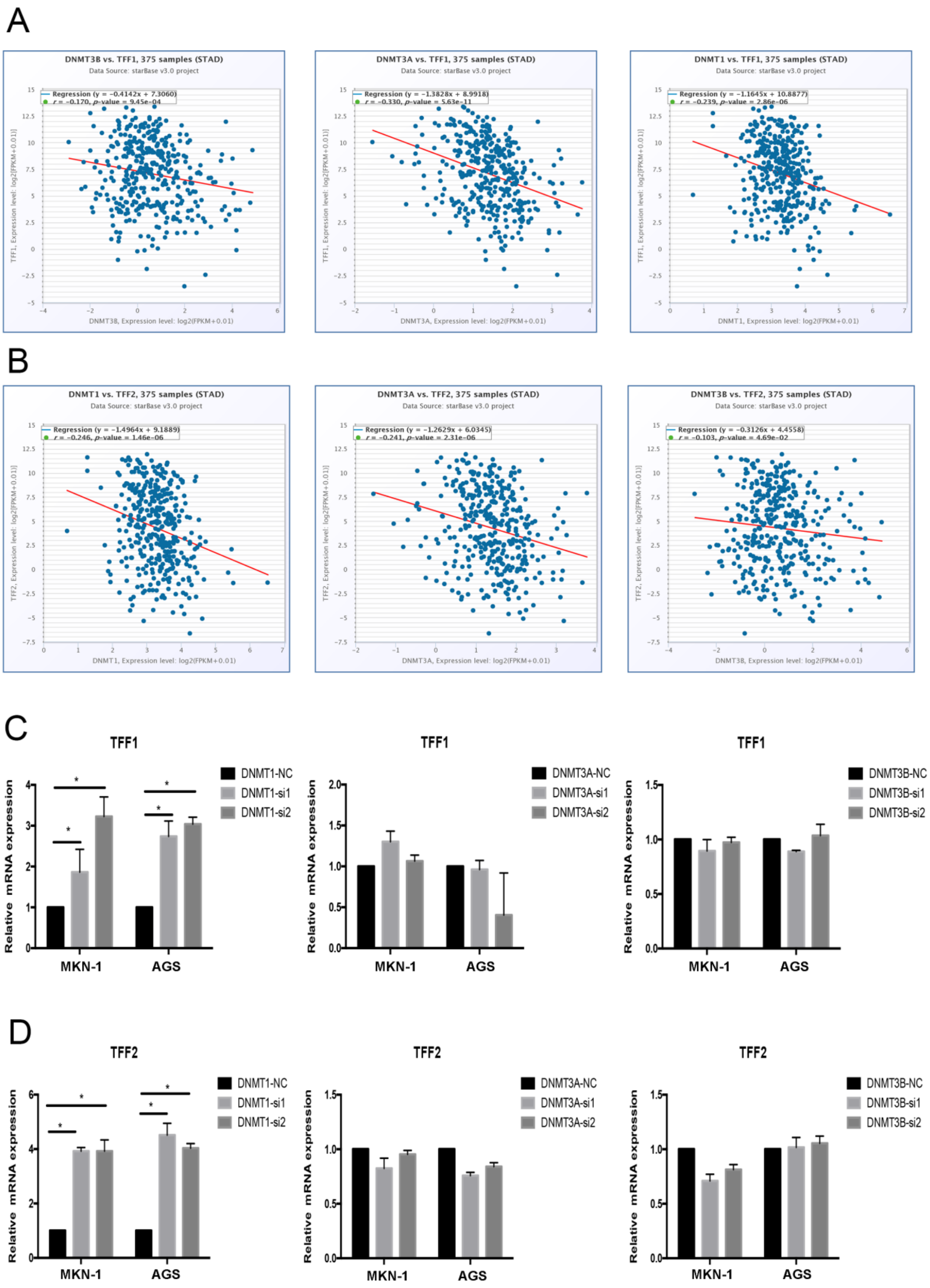

Figure S3 Through the Starbase database, the mRNA expression of TFF1/TFF2 were negatively correlated to the expression of DNMT1, DNMT3A, and DNMT3B (A and B). Then the knockdown of DNMT1 led to the upregulation of TFF1/TFF2 in gastric cancer cells, but not the DNMT3A and DNMT3B (C and D). (* *P*<0.05)

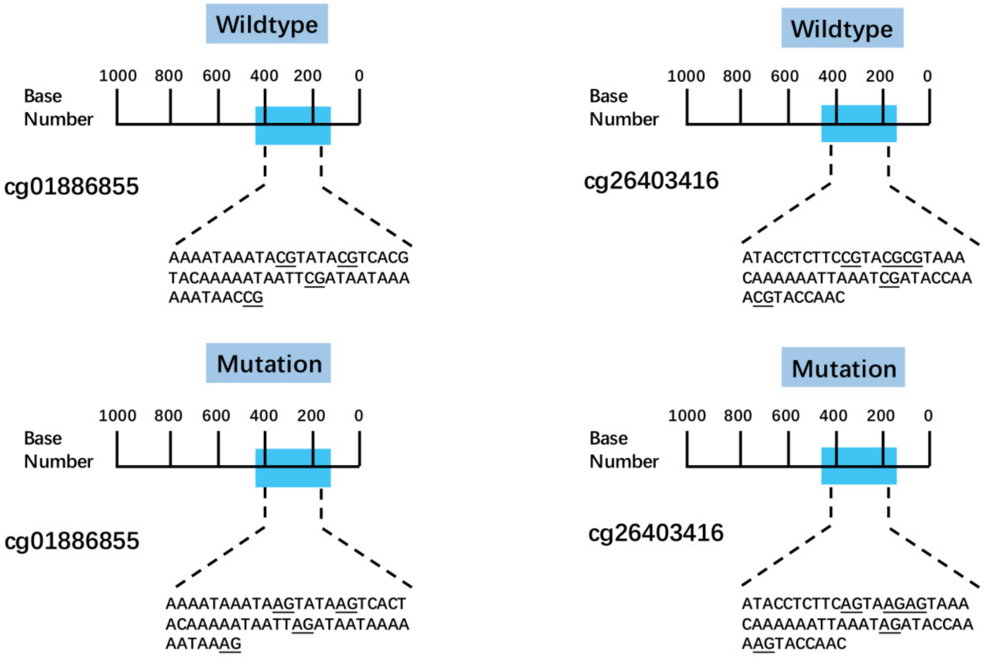

Figure S4 The wildtype and mutation sequence of cg01886835 CpG island site were shown in the left panel. And the wildtype and mutation sequence of cg26403416 CpG island site were shown in the right panel.
